# Chromosomal Instability in Acute Myeloid Leukemia

**DOI:** 10.3390/cancers13112655

**Published:** 2021-05-28

**Authors:** Mateus de Oliveira Lisboa, Paulo Roberto Slud Brofman, Ana Teresa Schmid-Braz, Aline Rangel-Pozzo, Sabine Mai

**Affiliations:** 1 Core for Cell Technology, School of Medicine, Pontifícia Universidade Católica do Paraná—PUCPR, Curitiba 80215-901, Paraná, Brazil; mol.lisboa@gmail.com (M.d.O.L.); paulo.brofman@pucpr.br (P.R.S.B.); 2 Hospital das Clínicas, Universidade Federal do Paraná, Curitiba 80060-240, Paraná, Brazil; ana.braz@hc.ufpr.br; 3 Department of Physiology and Pathophysiology, University of Manitoba, Cell Biology, CancerCare Manitoba Research Institute, Winnipeg, MB R3C 2B7, Canada

**Keywords:** chromosomal instability, acute myeloid leukemia, cytogenetic heterogeneity, aneuploidy, complex karyotype, *TP53*, centrosome dysfunction, *MYC*, telomere dysfunction, therapeutic targets, aging, synthetic lethality

## Abstract

**Simple Summary:**

Chromosome instability (CIN) is an increased rate where chromosome acquire alterations due to errors in cell division. CIN creates genetic and cytogenetic diversity and is a common feature in hematological malignancies such as acute myeloid leukemia (AML). Low to moderate levels of CIN seems to be well tolerated and can promote cancer proliferation, genetic diversity, and tumor evolution. However, high levels of CIN seems to be lethal, where enhancing CIN could improve AML treatment. However, little is known about CIN in AML. Our review focus on CIN studies in AML, their prognostic results, as well as the use of CIN as a therapeutic target in AML.

**Abstract:**

Chromosomal instability (CIN), the increasing rate in which cells acquire new chromosomal alterations, is one of the hallmarks of cancer. Many studies highlighted CIN as an important mechanism in the origin, progression, and relapse of acute myeloid leukemia (AML). The ambivalent feature of CIN as a cancer-promoting or cancer-suppressing mechanism might explain the prognostic variability. The latter, however, is described in very few studies. This review highlights the important CIN mechanisms in AML, showing that CIN signatures can occur largely in all the three major AML types (de novo AML, secondary-AML, and therapy-related-AML). CIN features in AML could also be age-related and reflect the heterogeneity of the disease. Although most of these abnormalities show an adverse prognostic value, they also offer a strong new perspective on personalized therapy approaches, which goes beyond assessing CIN in vitro in patient tumor samples to predict prognosis. Current and emerging AML therapies are exploring CIN to improve AML treatment, which includes blocking CIN or increasing CIN beyond the limit threshold to induce cell death. We argue that the characterization of CIN features, not included yet in the routine diagnostic of AML patients, might provide a better stratification of patients and be extended to a more personalized therapeutic approach.

## 1. Introduction

Since Boveri’s theory that chromosome abnormalities promote cancer, studies have attempted to elucidate the mechanisms behind the origins of chromosomal aberrations [1]. Chromosomal instability (CIN) is the increasing rate in which cells acquire new chromosomal alterations. Depending on the type of abnormalities, it can be classified into numerical CIN (nCIN), characterized by chromosome gains and losses, and structural CIN (sCIN) represented by chromosome translocations [2]. Importantly, CIN is one of the cancer hallmarks [3]. CIN can promote selective advantage to cancer cells by increasing the probability of novel chromosomal abnormalities, which can change the expression profile of the genes regulating cell division and differentiation, resulting in high proliferation rates [3,4]. Recent studies have shown a deep relationship of CIN with the origin, progression, and relapse in many cancers [5,6,7,8]. 

CIN not only occurs as a tumor-promotor mechanism but also as a tumor-suppressor mechanism. This observation comes from the evidence showing that different levels of CIN lead to distinct outcomes. Moderate or low levels of CIN are associated with increased rates of genetic cancer-promoting features. On the other hand, extreme levels of CIN could lead to decreased cell fitness or apoptosis [9]. The levels of CIN and the sites in which it occurs can also indicate different outcomes [10]. Therefore, CIN features not only could refine risk stratifications but also opens opportunities for new therapeutic approaches in cancer [9,11,12].

**Figure 1:**
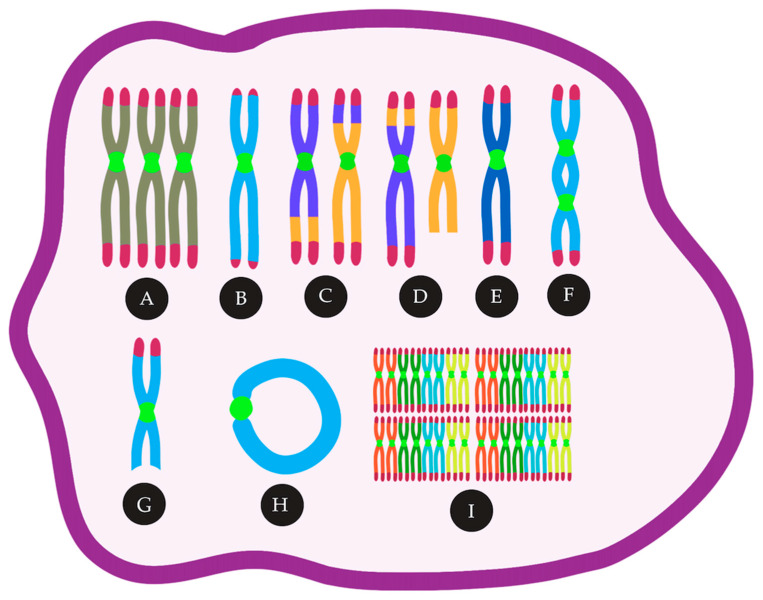
CIN in AML can lead to many cytogenetic abnormalities, such as (A) trisomies, (B) telomere loss, (C) reciprocal translocations, (D) unbalanced translocations, (E) monosomies, (F) Dicentric chromosomes, (G) deletions, (H) ring chromosomes, (I) polyploidy.

The current models for CIN involve telomere dysfunction, defective spindle assembly, sister chromatid cohesion, DNA double-strand breaks (DSB) repair, genes involved in the cell cycle, and epigenetic regulators. These CIN mechanisms and their signatures can be largely found in acute myeloid leukemia (AML), a heterogeneous disease characterized by abnormal proliferation and accumulation of myeloid precursor cells in the bone marrow [13]. AML can be classified as de novo AML, secondary AML (s-AML), whose origin is from a prior hematologic disease, and therapy-related AML (t-AML), which arises as a result of exposure to alkalizing agents, irradiation, and other factors associated to prior therapy [14,15]. Regardless of the classification, approximately 55% of AML patients show chromosomal abnormalities [16]. Cytogenetic abnormalities in AML are an important prognostic factor and are used for risk-stratification and guide treatment definition [17,18]. For example, a complex karyotype (CK) is associated with poor prognosis [19]. In older patients (≥60 years), only 10–44% of those with ≥3 cytogenetic abnormalities achieve complete remission (CR) after therapy, and for those with ≥5 chromosome abnormalities, the CR rates are significantly lower (7–26%) [20,21,22,23,24,25]. In this review, we will focus on the mechanisms associated with CIN resulting in cytogenetic abnormalities (Summarized in Figure 1), their prognostic impact, and the use of CIN as a target among the AML types. 

## 2. Mechanisms and Consequences of CIN 

### 2.1. Aneuploidy and CIN in AML, Mechanism and Consequence — An Inter-Relationship

One type of nCIN is aneuploidy, characterized by an abnormal number of chromosomes in the cell, which is a result of chromosome mis-segregation errors [26]. These errors can occur when the chromosomes fail to attach correctly to the mitotic spindle [27]. CIN and aneuploidy are not synonymous [28]. Although CIN is defined as the increasing rate in which cells acquire new chromosomal alterations, aneuploidy is associated to the abnormal number of chromosomes in the karyotype at a specific point in time [29]. Aneuploidy can also be found after clonal expansion, and this is not implying that the same cell will acquire new chromosomal alterations in every cell division. That is the case of constitutional trisomies, such as trisomy 21 or Down’s syndrome, which presents aneuploidy but not CIN [30]. However, patients with trisomy of the 21 have a predisposition to cancer, such as hematologic malignancies [31]. In AML, aneuploidy is present in more than 20% of cases [17,32,33,34] and is related to poor prognosis [17,33,35,36,37].

A key gene to maintain diploid karyotype in normal cells is *TP53* [38]. This protein regulates cellular differentiation, cell cycle arrest, and DNA repair preventing genomic instability [39,40]. Mutations in *TP53* arise before or after the first aneuploidy event [41,42] These mutations are also considered an early leukemogenic event in preleukemic stem cells [43,44]. Alteration in *TP53* may result in the continued proliferation of aneuploidy cells or can trigger off apoptosis [45]. *TP53* has a role in suppressing nCIN and sCIN by inducing apoptosis in cells that have a long pause in the mitotic checkpoint, which indicates DNA damage [46]. Mutations in tumor suppressor genes comprise 16% of AML patients, and *TP53* is one of them [34]. The incidence of *TP53* mutations in AML varies between 10% and 15% [34,47,48]. In general, *TP53* mutations are highly present in AML patients with CK (60%) [47,49,50,51].

Interesting, the incidence of *TP53* mutations in AML is low in comparison with other cancer types. This observation highlights that additional mechanisms are affecting p53 function for AML patients [52]. Many pathways have been proposed for non-mutational wtp53 inactivation in AML. Mdm4, a p53 negative regulator, is overexpressed in 10% of cases in wtp53 AML with CK [53,54]. Li et al. (2014) described that Mdm4 overexpression was associated with aneuploidy or polyploidy, showing an important link between Mdm4 overexpression, wtp53 inhibition and CIN in AML [53,54]. Additionally, Mdm2 overexpression, another p53 negative regulator, is seen in more than 50% of wtp53 AML patients [55,56,57]. Together with Mdm2 and Mdm4 overexpression, ARF down-regulation, deregulated post-translational modifications, and nuclear-cytoplasmic microRNAs were also described as non-mutational wtp53 inactivation in AML [52]. Cazzola et al. (2019) demonstrated that AML cells *TP53*-knockout have CIN phenotype and karyotype heterogeneity [58]. The deletion of 5q, a chromosomal region containing many protein-encoding genes associated with hematopoietic differentiation, together with *TP53* mutations, can promote genome and chromosomal instability to block normal hematopoietic differentiation and is significantly associated with CK and poor prognostic [59,60,61]. In addition, 5q deletion, which leads to the haploinsufficiency of the genes involved in cell cycle control, as a sole chromosomal abnormality, is rare in AML patients. However, together with *TP53,* this alteration is frequently found in AML patients with CK (60–69%) [62,63,64]. Together, these observations show a strong association between CK and the deletion of checkpoint genes (*TP53* and the ones located at 5q), suggesting an important role of dysfunctional cell cycle checkpoint in AML.

### 2.2. Chromosome Segregation Errors

#### 2.2.1. Defects in the Spindle Assembly Checkpoint (SAC)

Jin and Burkard (2018) have associated CIN in AML patients with defects in the spindle assembly checkpoint (SAC). SAC is a mitotic checkpoint mechanism used to prevent transition to anaphase when there is an error on the kinetochore-microtubules attachments [65,66]. When SAC is malfunctioning, cells without proper spindle attachments can bypass anaphase checkpoints and divide [1]. The inactivation of the entire mitotic checkpoint can generate chromosome mis-segregation leading to CIN or cell death [67,68] in various cancers [69,70,71]. 

Mosaic variegated aneuploidy (MVA) is a rare human chromosomal instability disorder. Patients with MVA present germline mutations in the mitotic checkpoint components and 25% of the cells show aneuploidy [72]. The most relevant mutation is in the spindle checkpoint gene *BUB1B,* where the SAC protein BubR1 is expressed. BubR1 stabilizes the kinetochore-microtubule and corrects the proper chromosome positioning. It prevents cell division until forming an appropriate bi-oriented mitotic spindle [73]. In MVA, mutations in *BUB1B* leads to CIN with consecutive constitutional mosaicism for chromosomal gains and losses and subsequent predisposition to several types of cancer [74]. In AML, Schnerch et al. (2012) suggested a role of SAC insufficiency in the pathogenesis and progression of patients with a CK [75]. Since *BUBR1* is an anaphase-promoting complex (APC/C) inhibitor gene, AML cells with defects in this gene usually allow chromosomal alterations to bypass mitosis [76]. 

The most common chromosomal abnormality found in AML is t(8;21)(q22;q22) [77]. Boyapati et al. (2007) demonstrated in cell lines that the resulting fusion protein t(8;21) from AML impairs the spindle checkpoint and promotes aneuploidy [78]. Nucleoporin 98 gene (*NUP98*) is another gene associated with SAC defects in AML. NUP98 regulates the timely destruction of securin by APC/C [79]. Cells with *NUP98* translocation contain aberrant securin (a regulatory protein of the metaphase-anaphase transition), leading to aneuploidy [80]. Another APC/C protein with decreased expression in AML is *Cdh1*, an antagonist regulator of SAC (which activates and mediates securin degradation). Therefore, the high number of SAC alterations in AML cells can be associated with other dysfunctional chromosome segregation features. 

#### 2.2.2. Cohesion Defects

Sister chromatids are kept together until the proper formation of the bipolar spindle. If cohesion between sister chromatids is lost, chromosomes mis-segregate [81]. Cohesin defects, which is a protein complex mediating sister chromatid cohesion, are also associated with aneuploidy and CIN [82,83,84,85]. Ley et al. (2013), using whole-exome sequencing, showed mutations in cohesin complex genes in 13% of *de novo* AML patients [34]. However, cohesin gene mutations did not show a prognostic impact in AML. This is probably due to the co-existence of cohesin complex mutations with *NPM1* (Nucleophosmin 1) mutations [86]. Interestingly, the co-existence of both mutations is associated with a favorable prognosis and normal karyotype in AML [87,88]. *NPM1* mutation results in the inactivation of the nuclear factor-κB (NF-κB) in the cytoplasm [89]. Since activation of NF-κB provides drug resistance to chemotherapy drugs in AML, the association between cohesion and *NPM1* mutations leads to favorable prognostic and chemosensitivity. 

#### 2.2.3. Centrosome Dysfunction and Assembly of Multipolar Mitotic Spindles

Another important mechanism related to CIN is centrosome dysfunction [90,91,92,93,94]. Dysfunctional centrosomes are characterized by the presence of aberrant centrosomes numbers, imbalances in centrosome-associated proteins expression, centrosome structural abnormalities, and alterations in the clustering of centrosomal components [95]. Cells presenting centrosome defects show the formation of multipolar mitotic spindles (cells with multiple centrosomes) [91,96]. Neben et al. (2003) have shown an association between abnormal centrosomes and the presence of cytogenetic alterations in AML. Interestingly, centrosome dysfunction allowed stratification into cytogenetic risk groups, where higher numbers of centrosome alterations were related to an increased adverse prognosis. The authors also suggested centrosome aberrations and multipolar mitotic spindles as the cause of numerical chromosome alterations in AML patients [97].

The aurora kinases, a family of serine-threonine protein kinases, have a key role in centrosome dynamics, mitotic spindle, and mitotic centrosomes [98]. Two common types of these proteins are Aurora A and Aurora B. Aurora A is active during the late S and early G2 phase and ensures a proper spindle assembly and chromosome alignment during mitosis [99]. On the other hand, Aurora B functions as a protein complex (through the G2 phase) and is in charge of bipolar attachment of the spindle to the centromeres and correct segregation of the sister chromatids [98]. Aurora kinases A and B are overexpressed in AML CD34^+^ blast cells compared to CD34^+^ from normal individuals with no evidence of hematologic diseases [100,101,102]. Lucena-Araujo et al.(2010) reported that high expression of Aurora Kinases A and B was related to unfavorable cytogenetic abnormalities, represented by CK and high blasts count in AML patients [103]. Yang et al. (2013) outlined that AML blasts overexpressing Aurora A were chemotherapy resistant. Aurora A negatively regulates p53 and, due to the role of p53 in the induction of apoptosis, its downregulation allows cells to escape from apoptosis induced by chemotherapy in AML [100]. 

### 2.3. DNA Double-strand Breaks

DSBs can lead to translocations and DNA deletions [104,105,106]. One of the major causes of DSBs is a failure in the chromosome decatenation (disentanglement of the chromosomes) [107,108,109]. In normal cells, the decatenation checkpoint during the G2 phase delays the entry into mitosis until every chromosome is decatenated by the enzyme topoisomerase IIα (topo II) [109,110]. Wray et al. (2009) reported that AML cells that fail to arrest at the mitotic decatenation checkpoint continue to proliferate due to Metnase activity. Metnase (SETMAR) is a SET-transposase fusion protein that promotes non-homologous end-joining repair even in the presence of Topo IIα inhibitor [111]. Jacoby et al. (2014) showed that DSBs response is abnormal in myeloblasts from t-AML patients, and this feature was associated with trisomy 8. The authors suggested that the association between abnormal DSBs and trisomy 8 in AML is related to *MYC* overexpression [112]. *MYC* is a proto-oncogene located at the long arm of chromosome 8 (8q24). *MYC* deregulation leads to DNA damage [113], the induction of genomic instability and telomere dysfunction [114]. The induction of DNA damage through DSBs seems to occur through direct *MYC*-mediated suppression of the NHEJ (Non-homologous end-joining), an important pathway that repairs DSBs in normal cells [115,116,117].

Trisomy 8 can be found in the blood of normal individuals [118,119]. Grove & Vassiliou (2014) proposed that it may be one of the early AML leukemogenesis events [120]. Importantly, the gain of chromosome 8 is one of the most common chromosomal abnormalities in AML. It represents 30–40% of cases alone or in association with other cytogenetic abnormalities, and it is known as the most frequent gain of chromosome in AML patients with CK [121,122,123]. 

### 2.4. Telomere Dysfunction

Telomeres are TTAGGG repetitive sequences directly associated with capping proteins, shelterin proteins, that protect the ends of chromosomes [124]. The linear chromosome DNA ends have a 3′ single-stranded overhang, which prevents those sites from being recognized as DSBs and activate DNA damage response pathways [125]. Telomere overhang length remains constant in healthy individuals over time [126,127,128]). However, in AML, Yan et al. (2013) reported that patients with abnormal karyotype presented shorter overhang length than those with normal karyotype [129]. The authors suggested the overhang length as an important prediction of poor prognosis in AML patients [129].

Telomeres become shorter at each cell division and, without telomerase, an enzyme that adds TTAGGG repetitive sequences to elongate the telomeres, cells undergo senescence [130]. The senescence occurs when telomeres become critically short, a phenomenon known as Hayflick limit, resulting in the cell cycle arrest. Nevertheless, cancer cells can bypass the telomere crisis through different mechanisms. Reactivation of telomerase is the most common mechanism to maintain telomere length, followed by the alternative mechanism of telomere lengthening (ALT) [131,132]. Telomerase reverse transcriptase (abbreviated as *TERT*, or *hTERT* in humans) is a catalytic subunit of the enzyme telomerase, which, together with the telomerase RNA component (*TERC*), comprises an important unit of the telomerase complex [133]. A decrease or loss of telomerase activity by mutations leads to telomere shortening, increasing the risk of CIN and, consequently, of cancer. The telomerase complex genes are frequently mutated in AML [134,135].

Swiggers et al. (2006) demonstrated that critically short telomeres in blasts AML patients lead to nCIN. The authors reported an increased rate of loss or gain of chromosome parts after telomere shortening [136]. Hartmann et al. (2005) also supported the relationship between short telomeres and CIN in AML. In both studies, telomere length and *h*TERT expression correlated with chromosomal abnormalities in AML patients. They found that telomere length in mononuclear cells of AML patients was significantly reduced compared to controls (peripheral blood granulocytes from healthy individuals). In addition, patients with abnormal karyotype presented shorter telomeres than those with normal karyotype. In contrast, extremely short telomeres (median of -3,7 kb compared to healthy donors) were found in AML patients showing multiple chromosomal abnormalities. Furthermore, *h*TERT continued to be associated with an increase in the karyotype complexity [137].

Capraro et al. (2012) have also shown that an abnormal karyotype was associated with shorter telomeres and extremely low telomerase activity in AML [138]. Interestingly, dyskeratosis congenita (DC), a disease characterized by bone marrow failure, also presents short telomeres and shows a high predisposition to AML (with approximately 200-fold for AML compared to the general population) [139]. Therefore, telomere shortening is viewed as an important feature in AML and to be related to poor prognosis [134,137,140,141]. However, Warny et al. (2019) reported data not corroborating with these previous studies. Their interesting findings point out that telomere length in the bone marrow mononuclear cells was similar in size both at the moment of diagnosis and at relapse. They also observed that telomere length increased after chemotherapy-induced remission, but no prognostic association was found [142]. Warny et al. (2019) correlated telomere maintenance with telomerase. This is an interesting observation since elevated telomerase activity and *h*TERT expression were reported in 87% of AML patients in another study [143]. Swiggers et al. (2006) also observed high telomerase activity, except that AML patients with short telomeres presented high telomerase activity. In this case, high expression of TRF1, a protein that is a negative regulator of telomere length, was proposed to explain the co-presence of high telomerase activity and extremely short telomeres [136,144]. 

Telomeres are also responsible for preventing end-to-end fusions of chromosomes, one of the major mechanisms that can trigger both nCIN and sCIN [145]. Critically short telomeres and, consequently, telomere aggregates can result in fused chromosomes with two centromeres (dicentric chromosomes) [146]. During anaphase, the dicentric chromosomes form a bridge between the bipolar spindles and the centromeres of the sister chromatids pulled in opposite directions causing their breakage. Importantly, such breaks can occur in different places of the chromosome, not necessarily between fused chromatids. The contiguous repetition of this process gives rise to the phenomenon known as breakage-fusion-bridge (BFB) cycles, which is associated with CIN [147,148].

Dicentric chromosomes (DC) are one of the major signatures of telomere dysfunction. The incidence of dicentric chromosomes varies among AML types, but in general is present in 8–15% of all AML cases [149,150]. DCs are mainly found in CK (23%), where more than one DC is usually present [59,151]. DCs play an important role in oncogenesis, as demonstrated by Gascoigne and Cheeseman (2013). The authors showed that the occurrence of a single dicentric chromosome could contribute to tumor initiation in AML [152]. Furthermore, Sarova et al. (2016) related the presence of DC to MDS progression to AML, in which the transformation was characterized by the acquisition of more complex karyotypes [151]. 

### 2.5. Complex Chromosomal Rearrangements

Complex chromosome rearrangements (CCR) have been extensively reported in AML [153,154,155,156]. Various mechanisms have been suggested to the occurrence of this phenomenon (e.g., non-homologous end joining (NHEJ), replication-based mechanisms, BBF cycles, telomere dysfunction) [157]. Some authors have been using the term chromothripsis for the event where genetic material suffers an enormous clustered chromosomal rearrangement on specific regions of one or few chromosomes in a single cell cycle [158]. Since chromothripsis is not proven to be the cause of this phenomenon, here we will describe these abnormalities just as CCRs [157]. Rausch et al. (2012) showed that in their cohort of AML *TP53* mutated patients, ∼47% of cases presented CCRs. The occurrence of CCRs was associated with a poor prognosis [153]. Rücker et al. (2018) reported CCRs in 35% of AML patients with CK. In 85% of cases with CCRs presented mutated *TP53* [159]. Once more, this data highlights the role of dysfunctional *TP53* on CIN in AML. Hence, Fontana et al. (2018) have found an incidence of 6.6% CCRs in a large cohort of AML patients (N=395). It was also reported that AML cells with CCRs also presented signatures of CIN, such as *TP53* alteration, a higher mean of copy number alteration (CNA), CK, 5q deletion, alterations in DNA repair, and cell cycle. They also observed that AML cells with CCRs had marker chromosomes with the *MYC* gene [155]. Furthermore, Gao et al. (2020) reported that in AML-MRC with CCRs, this phenomenon was associated with a lower number of white blood cells and platelets and a higher degree of karyotypic complexity. The most involved chromosomes in CCRs were the chromosomes 8 and 11, resulting in the amplification of *MYC* (8q24.2) or lysine methyltransferase 2A (*KMT2A*) (11q23.3) [160]. L′Abbate et al. (2018) analyzed *MYC* amplicons in AML. Their results provide evidence that CCRs are not related to a single catastrophic event as the chromothripsis model describes it but rather to an accumulative evolution [161]. Marker chromosomes are rearranged chromosomes whose genetic origin cannot be verified by conventional banding cytogenetics techniques [162]. In AML, Bochtler et al. (2017) reported that marker chromosomes could arise from CCRs and predict adverse prognosis [154]. Marker chromosomes were also suggested to be a risk classification factor for AML with adverse cytogenetics [163]. 

### 2.6. Epigenetic Regulation

Abnormalities in the epigenetic regulator Tet methylcytosine dioxygenase 2 *(TET2)* and Enhancer of zeste homolog 2 (*EZH2*) could induce CIN through the deregulation of histone modifications, which alters the chromatin structure and affect gene expression [164,165,166]. Mutations in the *TET2* are among the most common mutations in AML [167,168,169]. *EZH2* is located in 7q36.1, a chromosomal region affected by the loss of chromosome 7 (-7) or deletion of 7q, which reduces its gene expression [170,171]. The -7 and deletion of 7q are highly associated with CK and adverse prognosis [50,172]. Wang et al. (2020) reported that *TET2* is hypermethylated (downregulated transcription) in 30% of AML patients. Alterations in the expression of *TET2* or *EZH2* also protects against apoptosis by an unknown mechanism [173]. Göllner et al. (2017) showed that *EZH2* loss of function induced resistance to multiple drugs in AML [174]. Interestingly, the expression levels of the genes *TET2* and *EZH2* were also positively correlated to the CIN *MAD2* and *CDC20* genes expression levels [173]. The protein mitotic arrest deficient 2 (Mad2) and cell division cycle protein 20 homologue (CDC20) overexpression and downregulation are frequently altered in many cancers and associated with CIN. Both proteins are essential for the mitotic checkpoint, in which they act together as an APC/C inhibitor, preventing aneuploidy and, consequently, CIN [1,175,176]. Schvartzman et al. (2011) have shown, in a p53 mutant tumor model, that wtp53 represses mad2 and its upregulation is necessary for CIN in AML [174]. Overexpression of *CDC20* is more present in aneuploid than euploid AML [177]. 

## 3. Clinical Considerations 

A summary of CIN features and the studies associating them to AML pathogenesis, evolution to worst outcomes, and relapse are shown in Table 1. It is important to mention that the incidence of AML increases with age (mean age of 75 years) [13]. An example of how age affects AML prognosis is observed in acute promyelocytic leukemia (APL), a subtype of AML characterized by the presence of t(15;17). Patients with APL present median age of 41–48 years, having a favorable prognosis and rare CK incidence (only 4% of the cases) [178,179]. CIN increases with age due to accumulation of genetic abnormalities, defective DNA damage response, and bone marrow loss of function (decreased repopulate ability of the hematopoietic system) [180,181]. Indeed CK, aneuploidy, telomere dysfunction, abnormal epigenetics, *MYC,* and *TP53* abnormalities are all age-related in AML [20,21,25,182,183,184]. The prognosis of sAML and tAML, in general, is poor when compared to *de novo* AML. However, the *de novo* AML patients older than 60 years of age and adverse cytogenetics present a similar prognosis of sAML and tAML [185]. The three AML types also shows differences in the number of CIN features found (Table 2).

Beyond assessing CIN in vitro in patient tumor samples to predict prognosis, current and emerging AML therapies are exploring CIN to improve AML treatment. Small-molecule inhibitors of aurora kinases are in phase I/II clinical trials for AML [210,211,213,214,215,216,217,218,219]. Yang et al. (2013) demonstrated that the proliferation of AML cells overexpressing Aurora A was significantly decreased by using aurora inhibitors such as alisertib (MLN8273), an oral aurora A kinase inhibitor. The authors noticed that the reduced proliferation was linked to a decrease in the self-renewal capability of AML blasts and apoptosis induction [100]. Besides, Kelly et al. (2012) reported that alisertib also enhanced cytarabine’s efficacy, a frontline chemotherapy medication used to treat AML. Cytarabine inhibits the DNA synthesis and, together with alisertib, generates intense stress response, which explains the better efficacy of the combination [238]. Recently, Brunner et al. (2018), in a phase II trial, showed that 67% of AML patients with adverse karyotype and 75% of those with *TP53* mutations achieved complete remission with the use of alisertib [207]. 

Moreover, Ikezoe et al. (2010) have shown that the induction of apoptosis by barasertib (AZD1152), an aurora B inhibitor, is dependent on functional p53. One of the consequences of using barasertib is the generation of polyploid cells. Aurora B inhibition leads to cells directly re-entering the S-phase without the cytokinesis. Barasertib aurora B inhibition in *wild-type* p53 cancer cells leads to increased p53 protein level and expression of p53 target genes to inhibit tumor growth [220,239]. Taken together, these results suggest that aurora kinases are promising targets for the elimination of chemotherapy-resistant AML blast cells since alterations in *TP53* are present in only 10–15% of AML patients. Interestingly, in acute megakaryocytic leukemia (AMKL), characterized by the presence of abnormal megakaryoblasts, aurora inhibitors can induce blasts differentiation due to their properties of inducing polyploidy (normal megakaryocytes are polyploidy cells). Aurora inhibitors in AMKL induce the upregulation of CD41 and CD42 expression, two characteristic markers of differentiated megakaryocytes [240,241].

With respect to telomere dysfunction, AML cells could be targeted by telomerase inhibitors [201,207]. Bruedigam et al. (2014) demonstrated that in xenografts of primary human AML, the pharmacological or genetic inhibition of telomerase targets AML cells, decreases leukemia progression, and detains relapse following chemotherapy [242]. The authors used Imetelstat, a potent and specific telomerase inhibitor under clinical trials in many cancers [243]. Rusbuldt et al. (2017) also showed promising results of Imetelstat in AML. Combination of this drug with Venetoclax, an approved *BCL-2* inhibitor used to treat chronic lymphocytic leukemia, showed a synergistic effect on apoptosis both in cell lines and patient samples in vitro, with a prolonged survival in xenograft models [244]. DiNardo et al. (2020) in a phase 3 trial, demonstrated that Venetoclax combined with azacitidine improved overall survival and remission when compared to those treated only with azacytidine [212]. *MYC* overexpression can also be a target, as was demonstrated in preclinical studies using molibresib (GSK525762), an orally bioavailable drug, which reduced c-MYC expression and its downstream transcriptional effects [245]. This drug is currently under investigation to treat AML patients [204]. *MYC* inhibition could overcome resistance to cytotoxic drugs in AML cells by promoting differentiation [202]. Another small molecule inhibitor that targets *MYC* is JQ1 [203]. JQ1 combined with all-trans retinoic acid (ATRA) synergistically suppresses AML cells proliferation [205]. The combination of ATRA and JQ1, which targets *MYC* and *hTERT*, could be useful especially for patients with telomere dysfunction [205]. 

Moreover, *TET2* function can be restored by using ascorbate, a potentially non-toxic therapy that promotes DNA demethylation, differentiation, and cell death in leukemic cells [223,246,247,248]. This approach enhances leukemic cells sensitivity to PARP inhibitors [223]. The use of ascorbate has been associated with better outcomes for AML patients with *TET2* mutations [224,249].

Regarding patients with mutations in *TP53*, a phase II study performed by Cluzeau et al. (2019) demonstrated that the combination of APR-246 (a drug that reactivates the mutated p53) with azacitidine (a DNA methyltransferase inhibitor whose cytotoxicity interferes with DNA synthesis) showed a response rate of 75% including 56% of complete remission (CR) [195,196]. Polo-like kinase 1(PLK1), one important mitotic regulator overexpressed in AML, could also be targetable [250,251,252]. Moison et al. (2012) have shown that PLK1 inhibitors induce apoptosis in mutated and wild-type *TP53* cells with complex karyotype in AML [188]. PLK1 inhibitors are in clinical studies for AML [253].

Targeting CIN as an approach to AML therapy is another attractive strategy, but some drugs could increase CIN beyond the “accepted” threshold and actually induce cell death. Jin & Burkard (2018) [65] measured CIN by verifying the chromosome mis-segregation frequency in AML samples and demonstrated that high levels of CIN were correlated with better overall survival. They also induced CIN with AZ3146 (an inhibitor of the Mps1 mitotic checkpoint kinase) in AML cell lines and showed that Mps1 inhibition induced a robust type I interferon (I IFN) response. This response is known to trigger the activation of many immune system cells, promote exposition of antigen, and enhance T-cell responses. Therefore, high CIN and Mps1 inhibitors seem to be promising therapies in AML [19]. The lack of I IFN response is known to cause resistance against chemotherapy in other cancers, which is overcome by supplying I IFN [254]. The use of type I interferon to treat AML is also suggested by other studies [255,256,257]. Furthermore, the role of high levels of CIN as a cancer suppressor has been reported on gastric, non-small cell lung, ovarian, and ER-negative breast cancers [258,259,260]. 

High degrees of CIN can also induce cell death through a mechanism known as synthetic lethality. Synthetic lethality is characterized by simultaneous mutations in two different genes, resulting in a significant decrease in cell fitness compared with the same mutations occurring independently [11,261]. Synthetic lethal approaches are already being applied to AML studies and show promising results. One of the main causes of drug resistance in AML is apoptosis evasion [262]. Pan et al. (2017), using AML resistant mouse models, demonstrated that synthetic lethality induced by the combination of *Bcl*-*2* inhibition (an anti-apoptotic protein) and p53 activation overcome apoptosis resistance in AML [263]. Leukemic cells with mutated *BRCA1* are more dependent on functional poly (ADP-ribose) polymerase (PARP) proteins than normal cells. They depend on PARP’s DNA repair role by homologous recombination (HR) [264]. PARP inhibitors can be used to selectively kill *BRCA1*- or *BRCA2*-mutated cells in AML *BRCA1* mutated patients (BRCA1 loss is reported in 12% of AML patients) [168]. Furthermore, defective HR also provides the potential use of PARP inhibitors targeting other key components of HR [265]. In AML, the *AML1-ETO* fusion, a product of the chromosomal translocation t(8;21) (q22;q22), results in the repression of genes that are essential to the DNA damage response [266,267]. Esposito et al. (2015) demonstrated, both in vitro and in vivo, that the suppression of HR transcriptional programs in *AML1-ETO* or *PML-RARa* cells leads to sensitivity to PARP inhibitors [268]. Faraoni et al. (2015) has shown in vitro that the use of the PARP inhibitor Olaparib induced cell death in cell lines with 11q23 deletion [269]. They also demonstrated that this drug selectively killed leukemic blasts, not affecting normal bone marrow CD34 cells [269].

## 4. Conclusions

**Figure 2:**
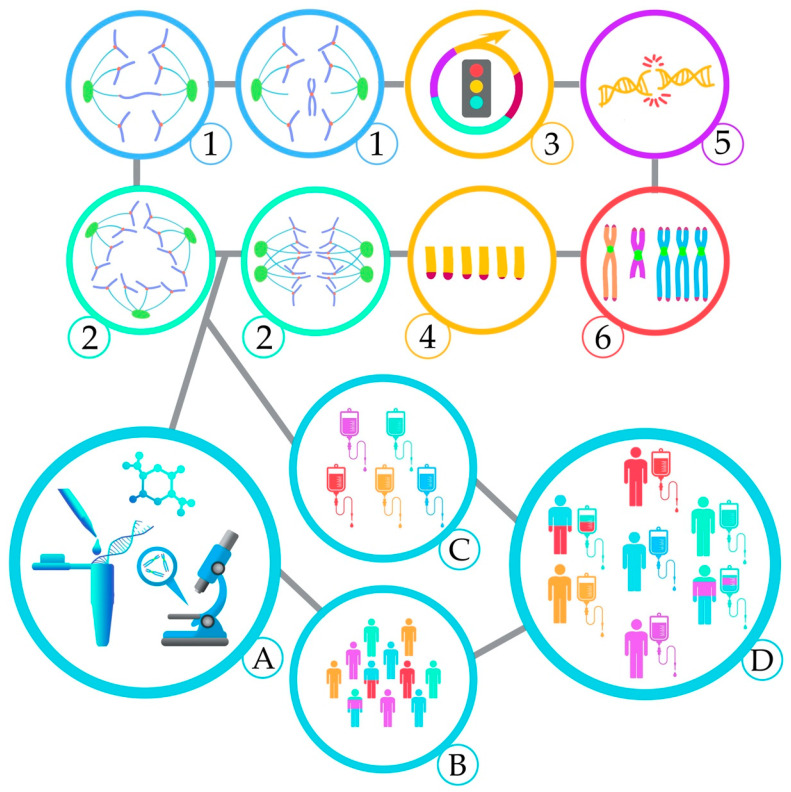
AML patients can present various CIN features, such as Chromosome mis-segregation (1); Multipolar spindles (2); Dysfunctional cell cycle checkpoints (3); Telomere dysfunction (4); Defective DNA damage response (5); Chromosomal abnormalities associated with CIN (6). These unique CIN markers could be used in clinical practice (A) for better stratification of patients with complex karyotype (B) and combine new target therapies (C) for personalized treatments (D).

The clinical outcome of AML is very heterogeneous and can range from few days of median survival to complete cure, depending on AML subgroups. In AML, the karyotype is the most powerful predictor of treatment outcome. Approximately 30% of cases of AML have an unfavorable karyotype and, if treated with conventional chemotherapy, a 5-year overall survival of 10% to 20% is expected. The best chance of cure for those patients seems to be an allogeneic transplant, but elderly patients are not eligible. Several studies had highlighted CIN as an important mechanism related to the origin, evolution, and relapse of AML. AML patients with CK can present various CIN features, such as defects in the spindle assembly checkpoint, centrosome dysfunction and assembly of multipolar mitotic spindles, defective DNA damage response, telomere dysfunction, and chromosomal abnormalities known to trigger CIN (Figure 2). CIN signatures can occur in all the AML ontogenies, but their incidence varies among *de novo* AML, sAML, and tAML. The higher incidence of chromosomal abnormalities found in tAML can be correlated to the prevalence of CIN features in this AML entity and results in the poor prognostic among the AML types. The CIN features in AML are strongly related to ageing, prior disease characterized by CIN, and previous exposure to cytotoxic therapy. Although most of these features show poor prognostic value, they also offer strong new perspectives on personalized therapeutic approaches to decrease the toxic effects of chemotherapy and relapse rates in AML patients. For each CIN feature occurring in AML, there are studies in different research phases showing promising results on targeting those specific mechanisms. Therefore, these studies open the perspective of combining multiple therapies based on the occurrence of the different CIN features individually or on their co-occurrence. The characterization of CIN features that are not in the current routine analyses of AML patients might provide a better stratification of patients. Studies based on the CIN approach with a more extensive characterization of CIN signatures may provide better therapy strategies, especially for those patients with a high risk of relapse or who do not respond properly to current chemotherapy. Future studies evaluating the association among the mechanisms cited here comprise venues for further exploration.

## Figures and Tables

**Table 1:** CIN features during AML course and drugs used in preclinical and clinical studies.

Features		Promising Agents ^1^
CK	[20,186,187]	PLK1 inhibitors [188]. In *TP53* mutated: APR-246 [189]
Aneuploidy	[190,191]	PLK1 inhibitors [188]. In *TP53* mutated: APR-246 [189]
*TP53*	[192,193,194]	APR-246 with azacitidine (AZA) [195,196]. APR-246 alone [189], APR-246 combined with azacitidine [197,198].
Abnormal 5q	[64,187,199,200]	PLK1 inhibitors [188]. In *TP53* mutated: APR-246 [189] APR-246 with azacitidine [195,196]. APR-246 alone [189]
*MYC* abnormalities	[201,202]	Molibresib (GSK525762) [203,204] JQ1 combined with All-trans retinoic acid (ATRA) [205] Dihydroergotamine (DHE) [206]
Trisomy 8	[118,119,120,121]	Molibresib (GSK525762) [203,204] JQ1 combined with All-trans retinoic acid (ATRA) [205] Dihydroergotamine (DHE) [206]
Telomere dysfunction	[136,144]	(hTERT) JQ1 combined with All-trans retinoic acid (ATRA) [205] Telomerase inhibitors [201,207].
Dicentric chromosomes (DCs)	[150,208]	PLK1 inhibitors [188]. In *TP53* mutated: APR-246 [189] APR-246 with azacitidine [195,196]. APR-246 alone [189]
Aurora Kinases	[100,101,102,103,209]	Small molecule inhibitors [210,211,212,213,214,215,216,217,218,219,220]
TET2 abnormalities	[173,221,222]	Decitabine [173]. Vitamin C combined with PARP inhibitor [223]. Ascorbate (Vitamin C) [224]
EZH2 abnormalities	[173,225]	Decitabine [173]

^1.^ Preclinical and clinical studies.

**Table 2:** Incidence of CIN features in different types of AML.

Features	*de novo* AML	sAML	tAML	References
Chromosomal abnormalities	47–51%	62.2%	75–92%	[185,226,227]
Adverse cytogenetics	19%	22%	39–40%	[185,226]
Complex karyotype (CK)	10–23%	18%	26- 40%	[24,25,226]
Abnormal 17p	5–11%	14%	14%	[33,226,228]
*TP53* Mutation	7–21%	15%	47%	[192,229]
−5 or 5q−	5–8%	7–14%	14–21%	[25,33,226,227]
Abnormal chromosome 7	2–7%	19%	10%	[25,33,226,230]
Telomere Length: TRF	No significant differences		ND	[141,231]
*MYC* overexpression	35%	27%	ND	[232]
+8	10–25%	25%	26%	[228,233]
Dicentric Chromosomes (DCs)	3%	ND	15%	[149]
DCs in CK cohorts	48%	59%	ND	[208]
*TET2* mutations	13–27%	24–29%	24%	[222,234,235]
*EZH2* abnormalities	2–4%	7–9%	8%	[229,236,237]

ND: not described.

## Data Availability

No new data were created or analyzed in this study. Data sharing is not applicable to this article.
